# Temporomandibular Joint Disk Displacements in Class II Malocclusion and Cervical Spine Alterations: Systematic Review and Report of a Hypodivergent Case with MRI Bone and Soft Tissue Changes

**DOI:** 10.3390/life12060908

**Published:** 2022-06-17

**Authors:** Oana Almășan, Andreea Kui, Ioana Duncea, Avram Manea, Smaranda Buduru

**Affiliations:** 1 Prosthetic Dentistry and Dental Materials Department, Iuliu Hatieganu University of Medicine and Pharmacy, 32 Clinicilor Street, 400006 Cluj-Napoca, Romania; oana.almasan@umfcluj.ro (O.A.); imduncea@yahoo.com (I.D.); smarandabudurudana@gmail.com (S.B.); 2 Department of Oral and Maxillofacial Surgery, Iuliu Hatieganu University of Medicine and Pharmacy, 400029 Cluj-Napoca, Romania; avram.manea@umfcluj.ro

**Keywords:** temporomandibular disorder, cervical pain, disk displacement, MRI, joint effusion

## Abstract

(1) Background: This study aimed to perform a literature review related to disk displacement (DD) in class II malocclusion or cervical vertebrae position alterations and to report a hypodivergent case with cervical pain and right anterolateral DD with reduction, left anterolateral DD with reduction, and left joint effusion. (2) Methods: A structured electronic search was conducted between March 2022 and April 2022, without time limits, following PRISMA guidelines, in the following databases: PubMed, Scopus, Embase and Cochrane; the terms “disc displacement”, “disk displacement”, “temporomandibular joint”, “class II malocclusion” and “cervical vertebrae” are searched. (3) Results: the following thirteen publications are included in this review: two prospective studies and eleven cross-sectional studies; for evaluating disk position, eight included publications used magnetic resonance imaging (MRI), whilst six studies used lateral cephalogram to determine craniofacial morphology and relationships between the cranial base, vertical skeletal pattern, maxilla and mandible. (4) Conclusions: although the literature still shows contradictory opinions, a relationship between temporomandibular disorders and cervical posture has been shown in the presented case as well as in the literature review.

## 1. Introduction

The functionality of the face and of the human body is dependent on the temporomandibular joints (TMJs) and their accompanying tissues [1]. Due to the multifactorial etiology and the high prevalence of temporomandibular disorders (TMDs), there is a continuous need for a redesigned, patient-centered, interprofessional approach to TMD treatment and prevention [2]. TMD refers to a series of musculoskeletal conditions that affect the TMJ, the masticatory muscles, as well as other tissues [3]. TMD is characterized by pain, joint noises, jaw movement limitation, muscle discomfort or joint sensitivity [4]. Clinical diagnosis of TMD is performed according to the Axis I of the Research Diagnostic Criteria for TMD (RDC/TMD), which validates the pain-related TMDs and the intra-articular disorders [4,5]. The definition of TMD kept on expanding, such as a workgroup formed by participants from various research fields, such as the American Academy of Orofacial Pain, the European Academy of Craniomandibular Disorders, the Australian and New Zealand Academy of Orofacial Pain, the International Headache Society, the Orofacial Pain SIG of the International Association for the Study of Pain, the International RDC/TMD Consortium Network of International Association for Dental Research, and the National Institute of Dental and Craniofacial Research finalized a classification comprising thirty-seven conditions, grouped into the following four categories, each of them having subgroups: temporomandibular joint disorders, masticatory muscle disorders, headache and associated structures [5].

The TMDs prevalence is reported worldwide as having various frequencies. Valesan et al., in a systematic review and meta-analysis, they have shown TMD to be prominent in about 31% of adults/elders and 11% of children/adolescents, with disk displacement without being the most frequent TMD as follows: in children/adolescents having a 25.9 percentage and in adults/elderly it has a 7.4% [6]. Wieckiewicz et al., in a study population among Polish urban adult subjects, found a percentage of 48.8 of TMDs, mostly disk displacement with reduction (47.9%) [7]. Qvintus et al. in 2020, assessing the prevalence of clinical signs and pain symptoms of TMDs and associated factors in 1577 adult Finns who took part in a health survey (2011), found that over 1/3 of the investigated population showed clinical signs of TMD, whereas only around a tenth had pain sensations [8].

Since it precisely illustrates the TMJ disk position relative to the condyle and prevents patient exposure to ionizing radiation, magnetic resonance imaging (MRI) is the gold standard for identifying TMJ disk displacement (DD) [9]. Regarding the relationships between TMD and postural and functional changes in the musculoskeletal system, it has been shown that mandibular condyle location in the articular fossa affected postural and functional alterations in the musculoskeletal system [10]. Moreover, significant postural alterations in the skull concerning the cervical vertebrae have been reported in TMD patients [11]. Pain caused by TMD can be associated with various sources of pain and a higher pain threshold [12].

The relationship between TMD and head and neck position has been studied [13]. The temporomandibular disorders (TMDs) consequences on craniocervical posture and hyoid bone position have been studied, even though results are still inconclusive [14]. Studies have shown that TMD is related to the position of the hyoid bone and the craniofacial anatomy and not to the craniocervical posture [15]. Characteristics of the cervical spine in patients with TMD show a lower pressure pain threshold and limited cervical range of motion [16]. However, other studies do not reveal a significant relationship between skeletal Class II and the cervical spine [17] or between TMD symptoms and changes in craniocervical posture [18].

Nowadays, in a study that investigated the psychoemotional well-being of both Israeli and Polish populations, it has been shown that the COVID-19 Pandemic had a negative impact, leading to an outbreak of TMDs or bruxism [19].

The relationship between skeletal pattern, head posture and TMD has been debated. There are differences in viewpoint in this area. Some studies support the association between them, whilst others do not. The existence of a functional relationship between TMJ DD, class II malocclusion and cervical spine position modifications is still controversial.

In light of the above-mentioned assertions, this study aimed to perform a systematic review related to TMJ DD in class II malocclusion subjects or cervical vertebrae position alterations and to report a hypodivergent case with cervical pain and right anterolateral disk displacement with reduction (DDR), left anterolateral DDR and left TMJ effusion. We aimed at binding this review with a particular clinical case due to the uniqueness of the clinical case, which hindered a distinct classification of the patient’s TMD, even when considering the latest TMD classification.

## 2. Materials and Methods

The review has been performed following the recommendations of the “Preferred Reporting Items for Systematic Reviews and Meta-Analyses Protocols (PRISMA) Statement” [20].

### 2.1. Eligibility Criteria

The inclusion criteria were temporomandibular disorder (TMD), class II malocclusion, disk displacement, cervical spine or vertebrae issues, articles in the English language.

The following exclusion criteria were considered: surgical intervention, TMJ arthritis, TMJ arthrosis, case reports, abstracts, editorials, letters to editors, communications, reviews and systematic reviews.

### 2.2. Information Sources

A structured electronic search was conducted between March 2022 and April 2022, with no time limits, in the following databases: PubMed, Scopus, Embase and Cochrane. MeSH and Emtree terms were used, where applicable. In addition, a handsearching of relevant studies was performed. All references were imported and organized in the Rayyan online software [21].

### 2.3. Search Strategy

Article selection was conducted in two phases. No time limit was set, nor any search filters or restrictions. To include all possible retrievable studies, a double search was performed. The first search included the terms “disc displacement” or “disk displacement”, “temporomandibular joint” and “class II malocclusion”. A second search looked for the terms “disc displacement” or “disk displacement”, and “temporomandibular joint” and “cervical vertebrae” of the above-mentioned terms. After retrieving all articles, two databases were created, using Rayyan online software that allowed to organize the publications and perform an independent, blind screening of the included studies. For reducing the selection bias, the “blind on” mode was applied. Two researchers (O.A. and I.D.) independently performed the search and scored the ratings. When in doubt about including a specific study, the researchers discussed between them and a third one was asked for debate (A.K.). All references were managed with Zotero software version 6.0.6 (Roy Rosenzweig Center for History and New Media, Fairfax, VA, USA) [22].

## 3. Results

### 3.1. Data Collection

A total of four hundred forty-nine articles were enrolled after applying the search strategy. After the elimination of the duplicates, three hundred fifty-seven articles were considered for screening. During the initial phase, the included studies were selected based on their title and abstract and their relationship to the research question. The screening process generated fifty-four articles for retrieval. The remaining articles were retrieved in full text and assessed for eligibility. A total of sixteen reports were assessed for eligibility. These were evaluated based on the inclusion criteria. Any disagreements were resolved by discussion and by consultation with a third one (A.K.). After a full-text reading of the remaining articles, thirteen articles were assessed for eligibility. Finally, a total of thirteen publications were included in this review.

**Figure 1:**
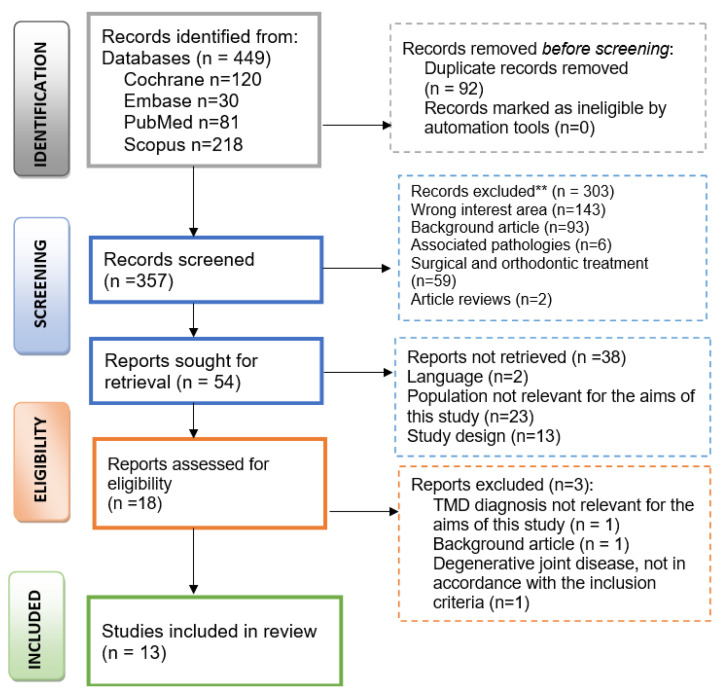
PRISMA flow diagram of the selection process. ** Records excluded by humans.

The selection process, along with the inclusion decision, is shown in Figure 1, the PRISMA flow diagram [23].

### 3.2. Description of the Studies and Analysis

The following data was extracted from the following articles: (1) authors and year of publication; (2) type of publication; (3) number of studied subjects; (4) mean age of subjects; (4) TMD diagnostic method; (5) outcome; (6) author’s conclusion.

The main characteristics of the studies that were considered in this review are summarized in Table 1.

### 3.3. Case Report

A thirty-nine-year-old Caucasian female patient presented for treatment with a chief complaint of temporomandibular disorder, associated with headaches and neck and shoulder pain, with a duration of more than six months.

The patient had a history of car collisions thirty-one years prior, in which she suffered multiple traumas, including cranial bone fissures (left temporal and occipital region). She also reported teeth clenching and night bruxism for more than ten years and over-the-counter medication used for headaches and muscle tenderness. The patient was also diagnosed with chronic rhinitis and bilateral chronic maxillary sinusitis by an otorhinolaryngologist, who preliminarily diagnosed fibromyalgia and TMD as well; therefore, she was referred to an orthodontist for evaluation and treatment.

The subsequent clinical examination was performed by both an orthodontist and a TMD specialist, with over twenty years of experience. During history taking, the patient also revealed pain in the right temporomandibular area with TMJ clicking and popping and uncomfortable jaw motions. The patient also reported right throat soreness and neck, shoulder and back pain. She also mentioned associated tinnitus in the right ear.

After filling out the Kinnie-Funt Chief Complaint Visual Index for Head, Neck and Facial Pain and TMJ dysfunction [35,36], we found that she ranked the following as her top three complaints: right sore throat without infection; “migraine”—type headache; tired, sore, neck muscles. The patient also noted additional complaints such as the following: inability to open the mouth smoothly and evenly; clicking, popping temporomandibular joints, pain in jaw muscles, balance problems (“vertigo”), ear pain without infection, upper and lower back pain. Neck pain was rated 7/10 on a VAS pain scale [37].

After performing a clinical examination, RDC/TMD criteria indicated a clinical diagnosis of bilateral disk displacement with a reduction [4,38].

The patient was referred to perform a lateral cephalogram along with magnetic resonance imaging (MRI) of the temporomandibular joints.

The lateral cephalogram showed a hypodivergent class II skeletal pattern, with a small anterior facial height, skeletal deep bite tendency and normal overbite and overjet (Table 2).

**Figure 2:**
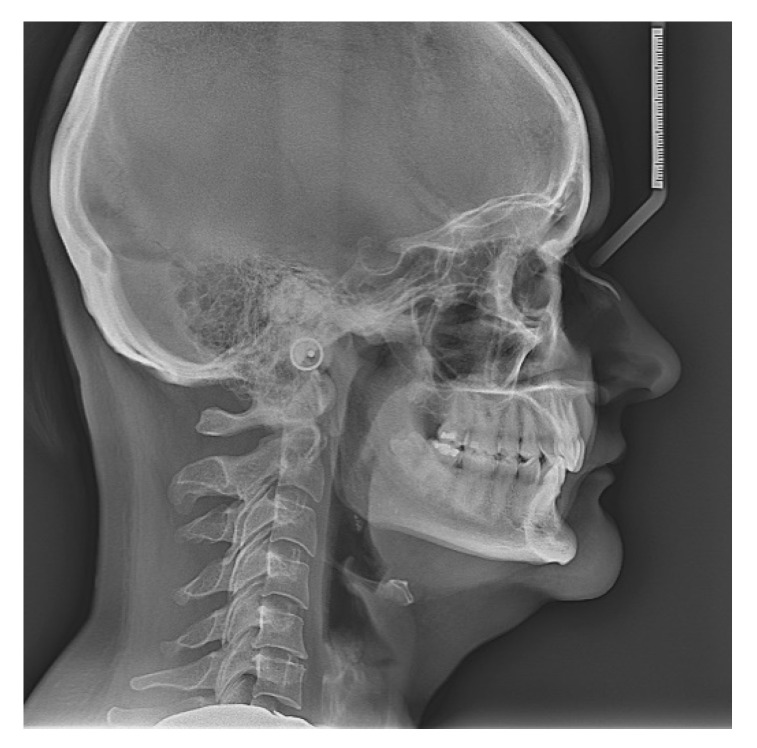
Lateral cephalogram.

The lateral cephalogram of the patient is shown in Figure 2.

**Figure 3:**
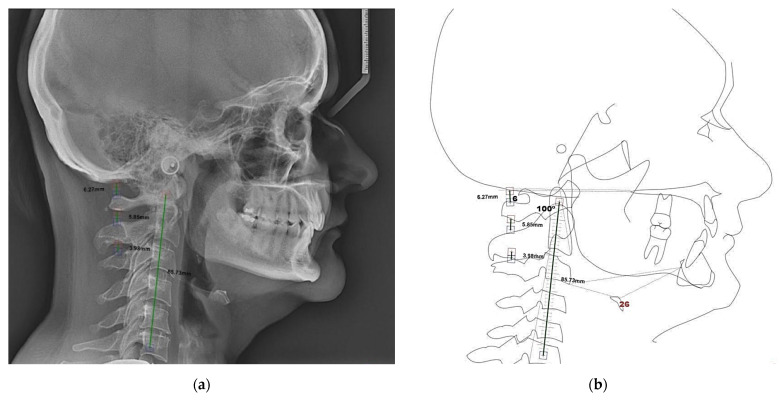
Rocabado analysis: (**a**) Measurement of the distances between C0–C1, C1–C2, C2–C3, hyoid triangle, craniovertebral angle, occipital-atlas angle; (**b**) Schematic representation of the analysis.

The craniocervical posture was evaluated by performing the Rocabado analysis [39]. In the following, we evaluated: the craniovertebral angle, the hyoid bone position, and the main vertebrae distances. The distance between cervical vertebrae was evaluated as follows: suboccipital space (the distance from the occiput to the first cervical vertebra; cranium—atlas distance, C0–C1), atlas–axis distance (C1–C2), the distance between the axis and third vertebrae (C2–C3). The following values were found: hyoid bone position of 25.9 mm, craniovertebral angle of 100°, and occipital-atlas angle of 6.1°, and for the following vertebrae distances: C0–C1: 6.27 mm, C1–C2: 5.85 mm, C2–C3: 3.98 mm (Figure 3).

The Rocabado analysis showed a modified hyoid bone position as well as decreased space between C1–C2 and C2–C3 vertebrae; moreover, the C2 vertebrae showed a rotation and the cervical spine had a vertical orientation.

**Figure 4:**
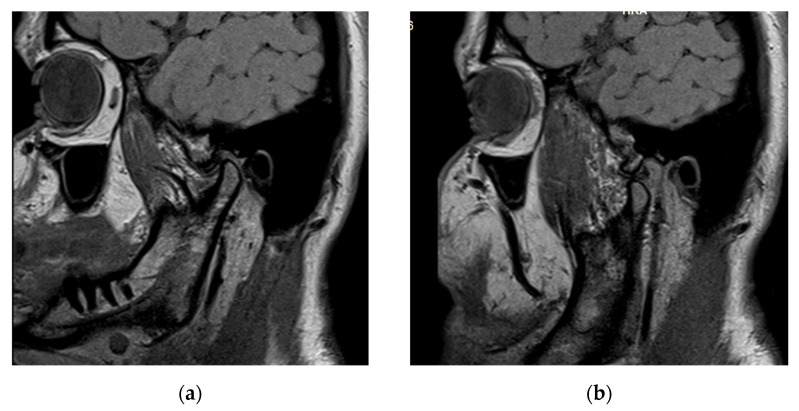
Sagittal (**a**) closed and (**b**) open mouth proton density MRI of the right joint: anterior disk displacement with reduction (DDR).

On sagittal proton density MRI of the right joint, in an open mouth position, the posterior border of the distal band was positioned anteriorly to the posterior slope of the articular eminence and the head of the condyle, indicating an anterior disk displacement (DD). In the closed moth position, the disk was centered over the condyle, showing a disk displacement with reduction (DDR, Figure 4).

**Figure 5:**
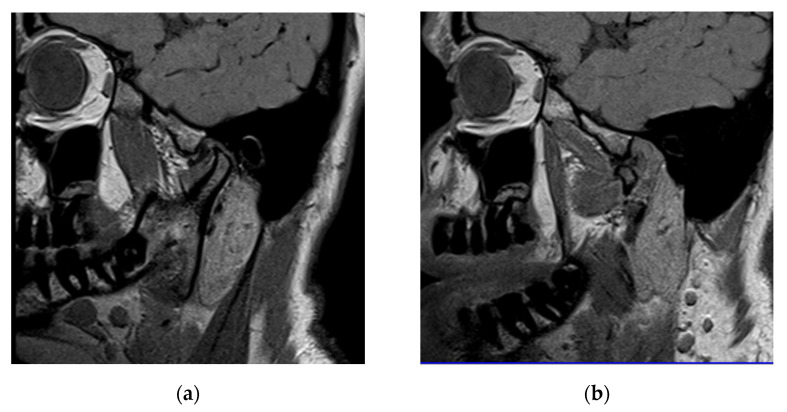
Sagittal (**a**) closed and (**b**) open mouth proton density MRI of the left joint: anterior disk displacement with reduction (DDR), condylar bone changes (flattened condyle, with posterolateral compression of the left condylar head, lateral resorption of the left condylar head with posterior position of the condyle in the articular fossa) and thickened posterior band disk shape.

Sagittal proton density MRI of the left joint showed in an open mouth position an anterior disk displacement (DD). In the closed moth position, the disk was recaptured, showing a disk displacement with reduction (DDR, Figure 5). Bone changes of the left condyle were found; a flattened condyle with posterolateral compression of the left condylar head and lateral resorption with posterior positioning of the condyle in the articular fossa. A thickened posterior band disk shape was also noted.

**Figure 6:**
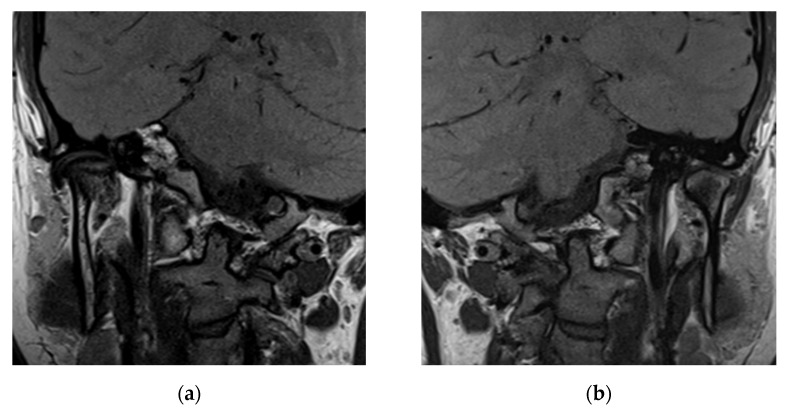
Coronal proton density MRI of the temporomandibular joints: (**a**) left and (**b**) right lateral (external) disk displacement with modified condyle shape.

In coronal MRI sequences, the right and left disk were found to be laterally displaced, as shown in Figure 6.

**Figure 7:**
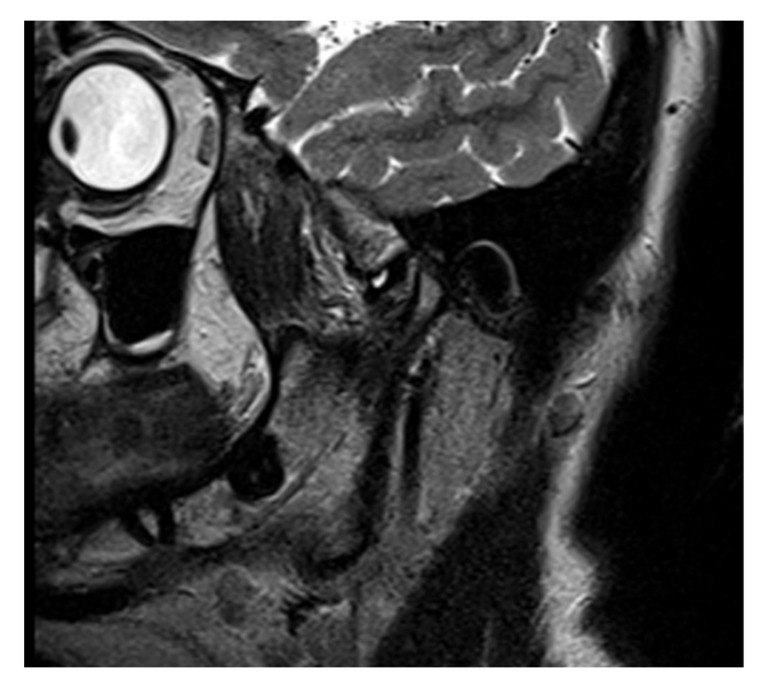
Sagittal oblique closed mouth position T2-weighted MRI of the left joint: joint effusion.

In sagittal T2 weight images, left joint effusion was found, as being an increased signal intensity in the joint space (Figure 7).

Based on the MRI examination, a diagnosis of right lateral disk displacement with reduction and left anterolateral disk displacement with reduction and left joint effusion was established.

## 4. Discussion

In this review were included a number of thirteen articles. Among these, two were prospective studies and eleven were cross-sectional studies.

A number of 1612 subjects resulting from the thirteen selected publications were included in this review. The majority of publications included young adult subjects, with ages ranging between 18 and 40 years; just a single study included young juvenile class II patients with anterior DDR [32]. Six of the studies included a control group and a study group. Most studies used the lateral cephalogram to determine the skeletal pattern [14,24,30,31,33].

Due to its excellent diagnostic accuracy, MRI is the gold standard in identifying TMJ disc position related to the condyle and articular eminence [40]. MRI investigation is also the standard reference in the diagnosis of inflammatory diseases of the TMJ [41] and can be also used for evaluating TMJ bone alterations [42]. For evaluating disk position, a number of eight included studies in this review used MRI images [14,24,26,29,30,31,32,33].

A number of six studies used lateral cephalogram to determine craniofacial morphology as well as relationships between the cranial base, vertical skeletal, maxillary and mandibular dental and soft tissue [14,17,24,26,30,31].

Flores et al. used lateral skull and cervical teleradiography for performing cephalometric measurements. Along with morphometric variables, the authors evaluated the posterior–inferior angle, atlanto-occipital functional space, hyoid triangle and depth of the cervical skull curvature [28]. The authors concluded that there is a link between morphological and functional features of the cervical spine in patients with TMD. D’Attilio et al. evaluated the cervical lordosis angle (CVT/EVT) in class II subjects with TMD and found a significant relationship between several cephalometric parameters and CVT/EVT, such as the following: maxillary and mandibular protrusion, mandibular length, overjet and overbite and significantly reduced cervical lordosis angle in TMD [26].

Di Giacomo et al., combined the standard cephalometric analysis with Rocabado’s approach [43] for determining other cervical characteristics as follows: the craniocervical angle, the C0–C1 vertebrae distance, the C1–C2 vertebrae distance, the hyoid bone position [17]. The authors suggested that functional modifications in the mandible may influence cervical spine evaluation, although they did not find a relationship between class II and cervical spine alterations. Matheus et al. evaluated the association between DD and skull position related to the cervical spine by examining the craniocervical angle, the suboccipital space between C0–C1, the cervical curvature and the position of the hyoid bone [33]. They, too, found no proven link between TMD and the evaluated parameters. In addition, Câmara-Souza et al., by studying the position of the hyoid bone, the craniocervical angle and the occiput–atlas distance, found no association between TMD and cervical posture [25]. On the contrary, Flores et al. concluded that the anatomical and functional characteristics of the cervical spine in patients with TMD are related [28]. De Farias Neto et al. also found an association between craniocervical angles and distances and TMD, namely, flexion of the first cervical vertebra as well as hyperlordosis of the cervical spine [27].

The objectives of the prospective studies included in this systematic review were to evaluate the influence of occlusal splint therapy on cervical spine pain and range of movement in patients with myofascial pain or DDR [34] and, respectively, to determine whether an anterior repositioning splint (ARS) can effectively treat anterior DDR in juvenile class II patients [32]. The mean treatment duration with an ARS reported by Ma et al. [32] was 11.5 months. The authors reported significantly improved pain and function, therefore a successful treatment. In addition, Walczynska-Dragon et al. showed significant improvements in TMJ function after three months of occlusal splint therapy, with 78 percent of the participants experiencing no DDR symptoms [34].

The relationship between head posture, cervical pain and occlusion has been intensively studied and debated. Neck pain may be associated with forward head posture [42,43,44] whereas Manfredini et al. state that occlusal and postural features and TMDs are not related [45], and Haralur et al. found an influence of the head posture on dynamic occlusal parameters [46].

Nevertheless, the biomechanical behavior of the TMJ is considered to be influenced by the head posture [47]. Inoue et al. studied the relationship between TMJ DD diagnosed by MRI and muscle pain patterns and showed that DD is possibly associated with ipsilateral muscle soreness [48]. Still, it has been shown that there is a relationship between improved head and cervical posture and the reduction of TMD symptoms [49].

It has been shown that craniofacial morphology and the cervical spine are interrelated structures [50,51]. Although there are relationships between occlusal variables and postural changes, there is little actual data to suggest a direct causal correlation [52]. An awkward posture is shown to be involved in neck pain [53] as well as the biomechanical posture of the cervical spine [54].

Although in the presented case we did not measure the Cobb’s angle for evaluating the cervical lordosis, it is obvious that the cervical spine has a vertical orientation by drawing the line connecting the second and seventh cervical vertebrae, sustained also by the modified hyoid bone position. The change in the depth of the cervical skull curvature, as described by Penning [55] and highlighted by Flores et al., was modified in TMD patients, implying the absence of the physiological lordosis of the cervical spine [28]. This is in accordance with our findings. The reduced hyoid triangle is encountered when the neck muscles are stretched and tensioned. The hyoid bone is a functional unit that allows mandibular and cervical dynamics [56]. The modifications of the hyoid triangle were related to TMD [24], which is in concordance with our findings, which showed a major increase in the hyoid triangle. Flores et al., have shown that in subjects with TMD the craniovertebral angle was decreased (below 96°) [28], whereas our patient had a craniovertebral angle of 100°, but with similar symptomatology.

The space between C2 and C3 vertebrae was decreased, also the C2 vertebrae showed a rotation, which could lead to transmitting an increased pressure on the spinal nerves. Concerning the functionality of the atlanto-occipital joint, involved in flexion and extension of the head, our findings are in concordance with the authors of de Farias Neto et al., who showed that in subjects with TMD there was an increased predisposition to flexion [27].

Greenbaum et al., when investigating the connection between TMDs and upper neck performance, have shown that subjects with pain-related TMD diagnoses are more likely to have considerable upper-neck hypomobility and poor muscle assets than patients with intra-articular TMDs [57]. On the contrary, our case report showed neck-related symptoms as well as bilateral disk displacement with reduction and left joint effusion, indicating an intracapsular disorder. Nevertheless, it has been reported that in patients with myogenic temporomandibular dysfunction, especially in women, there is a possibility that the higher cervical joints (C1–C2) are involved, the cervical range of motion and the extent of rotation during cervical flexion being reduced [58]. Moreover, Greenbaum et al. pointed out that cervicogenic headache was associated with pain-related TMDs, especially hypomobility and upper neck symptoms [59]. Our case also had upper cervical spine modifications, which could highlight the above-mentioned statements.

A debate relates to the changes in the vertical dimension of the head and changes in the craniocervical relationships. Solow and Tallgren found associations between craniofacial morphology and head posture [60], as well as Sharma et al. have found [61]. Liu et al. have described that subjects with skeletal class II patterns had a tendency to have a more extended head [62], with techniques of extension traction restoring lordosis in the cervical spine [63].

When Derwich et al., was studying craniovertebral and craniomandibular changes in patients with TMDs, have shown that the vertical and sagittal position of the mandible, as well as the width of the functional space between C1 and C2, were considerably influenced by combined occlusal splint therapy and physiotherapy [64].

McCormick et al. have shown that spinal cord compression may lead to neck pain [65]. Due to the fact that the head and neck complex are maintained in a neutral posture by the muscle forces [66], any disruption between the equilibrium of the muscles or the axial position of the vertebrae could lead to cervical and temporomandibular joint pain. Our presented case had a low anterior facial height and a hypodivergent profile and class II skeletal pattern, indicating a muscle imbalance of the neck muscles, leading to the pain symptoms. It has been shown that in children with class II malocclusion, low-intensity pulsed ultrasound (LIPU) associated with functional therapy may aid in mandible growth stimulation [67]. In adults, LIPU, due to its properties in soft tissue repair and bone regeneration, is used to stimulate mandibular condylar cartilage tissue regeneration and to decrease the progression and development of osteoarthritis [68]. At first, we considered LIPU in conjunction with the occlusal splint and physiotherapy to treat the joint effusion, but we decided to postpone this treatment method due to the patient’s lack of joint pain.

Obtaining correct facial proportions, controlling the factors that influence the hyoid bone position and maintaining the functionality of the mandible, as well as pain relief by myorelaxant agents, nonsteroidal anti-inflammatory drugs and physiotherapy/kinetotherapy, is vital in obtaining functional harmony. Occlusal splints may contribute to increasing the vertical dimension of occlusion, therefore aiding in symptom relief, being part of this complex treatment approach.

### Implications for Practice and Future Research

Based on the systematic review results and the reported case, there is evidence suggesting a correlation between cervical pain, head posture and temporomandibular disorders. The recommendations regarding therapy refer to conservative therapy as a first step, including pharmacologic treatments (non-steroidal anti-inflammatory drugs) associated with cognitive behavior therapy and biofeedback in order to improve short- and long-term pain management, as well as physical therapy [69]. Among the most commonly reported conservative therapies are custom-made occlusal splints and massage therapies, with additional methods being light and laser therapy or drugs [70].

Following a re-evaluation performed in two to four weeks, other conservative measures could be considered as well, such as relaxing therapies for muscle spasms and occlusal splints. Occlusal adjustments and orthognathic surgery, as well as joint surgery, might also be taken into consideration; however, surgical procedures (arthrocentesis, arthroscopy, diskectomy, condylotomy, total joint replacement) are rarely indicated in the case of TMDs; usually, they are chosen for correction of anatomic or articular abnormalities [71,72,73]. Surgical techniques, such as arthrocentesis, should be considered in refractory cases after a six-month period of splint treatment with no pain improvement or symptom relief. Due to the possible associations between head and neck pathology and TMD symptoms, a specialist in the cervical spine (physio/kineto therapist) with knowledge of the TMD area should be included in the workflow of this complex disease approach.

The relationship between TMD, head and neck posture and the skeletal pattern is still challenging. We tried to enlighten the connection between these, although the literature still debates contradictory opinions. We believe that a connection between them exists; however, which one relates to the other in terms of sequence, causality or influence is unclear. Correcting a malposition of the spine or a dental malocclusion still remains challenging in terms of TMD prevention.

Considering that craniocervical posture is related to facial characteristics and temporomandibular joint disorders, we emphasize the need for careful consideration of the muscle equilibrium of the head and neck, as it can have an impact on treatment outcome. We consider that the results of this review are relevant for practitioners, as they can reveal a possible association between skeletal pattern [74], cervical spine posture and TMD that may assist in treatment planning. We attempted to identify future areas of interest in the medical field that need more investigation. However, due to the limited methodological quality of the studies and the heterogeneity of the data, results should be interpreted with caution.

## 5. Conclusions

Based on the results found by performing the systematic review, we can conclude that the association between head position, cervical symptoms and occlusion has been investigated and debated intensively. Additionally, while the literature still shows contradictory opinions, a relationship between TMD and cervical posture has been revealed.

According to the literature, occlusal splint therapy can result in significant improvements in TMJ function. Occlusion, postural alterations, craniofacial morphology and TMD have been correlated. The findings revealed by reviewing the existing literature are consistent with the case report disclosed.

The lack of physiological lordosis, associated with modifications of the hyoid triangle and a decreased space between C2 and C3 vertebrae, may explain an over-pressure on the spinal nerves and the overall patient’s symptomatology. In this context, the literature suggests that occlusal splints, as part of this comprehensive therapeutic approach, may help to increase the vertical dimension of occlusion, therefore aiding in symptom relief.

Based on our findings, we believe that further research should be performed based on a higher number of subjects with TMD and cervical spine modifications, using the RDC/TMD protocol, cervical examination protocol and MRI.

There is a relationship between TMD and symptoms, as well as the cervical spine and occlusion. The treatment guidelines should include the cervical spine assessment as part of the initial reversible therapy and, in addition, postural treatment with a physiotherapist and a kinetotherapist, as well as splints for occlusion, without irreversible alterations to the dentition. The first treatment option aims to alleviate symptoms by addressing the spine while using splint therapy, with irreversible tooth changes being considered only afterward (orthodontics, prosthodontics). Other specialists are being considered as well, yet this approach may be included in the RDC/TMD protocol in the future.

## Figures and Tables

**Table 1:** Included studies.

Authors, Year of Publication	PT	Number of Studied Subjects	Mean Age of Subjects	TMD Diagnostic Method	Aim	Outcome	Conclusion
Jung-Sub An et al., 2015 [14]	CS	170 female orthodontic patients	24.5 ± 5.7 years (range from 17.0 to 50.8 years)	lateral cephalograms MRI 3 groups: bilateral DDR, bilateral DDwR, bilateral N	to evaluate craniocervical posture and hyoid bone position in patients with TMJ DD	− hyoid bone position in relation to craniofacial references was not significantly different among the TMJ disc displacement − extended craniocervical posture was significantly correlated with backward positioning and clockwise rotation of the mandible	“craniocervical posture is significantly influenced by TMJ disc displacement, which may be associated with a hyperdivergent skeletal pattern with a retrognathic mandible”
Ahn SJ et al., 2004 [24]	CS	58 women	>18 years	lateral cephalogram class II malocclusions MRI 3 groups: DDR, DDwR, N	to determine the association between the progression of ID and alteration in the dentofacial morphology	− decrease in posterior facial height, a decrease in ramus height, and backward rotation and retruded position of the mandible in the subjects with ID of the TMJ	“lower posterior facial height and ramus height, backward rotation of ramus and mandible, and relative protrusion of upper and lower lips were found in the patients with ID of the TMJ. These changes became increasingly severe as ID progressed to DDwR ID of the TMJ might induce dentofacial changes”
Câmara-Souza MB et al., 2017 [25]	CS	80 randomly selected students, 28 patients with TMD, 52 with no TMD 54 females and 26 males	18–30 years	lateral radiographs clinical diagnosis of TMD (RDC/TMD)	to evaluate the relationship between TMD and craniocervical posture the position of the hyoid bone, the craniocervical angle and the occiput–atlas distance	− 62% subjects modification on hyoid bone position − 47.5% extension or flexion of the head − 42.5% anterior rotation − no association between TMD and occiput–atlas distance, position of the hyoid bone, craniocervical angle	“no relationship can be found between craniocervical posture in the sagittal plane and the presence of TMD”
D’Attilio M. et al., 2004 [26]	CS	study group: 50 females with TMD (DD), class II malocclusion control group: 50 females without TMD	25–35 years 28.9 years average (SD = 3.2)	lateral cephalograms CVT/EVT angle MRI standardized TMJ clinical examination	to evaluate the existence of a relationship between morphological features of subjects with TMJ DD and CVT/EVT angle	postural variables of the cervical column were associated mandibular length, mandibular divergence and overjet	“in TMJ DD, an increase of the CVT/EVT angle was associated with an increase of mandibular and maxillary protrusion; a decrease of mandibular length; an increase in overjet; an increase in mandibular divergence; and a decreased overbite”
de Farias Neto JP, et al., 2010 [27]	CS	23 subjects 2 groups: (1) no TMD: 11 individuals; (2) TMD group: 12 subjects	from 18 to 30 years	clinical examination RDC/TMD self-reported symptoms questionnaire radiograph of the cervical spine	to compare the craniocervical angles and distances between TMD and no TMD	− reduced plane atlas angle in TMD (which verifies the craniocervical posture) suggesting a suggests a flexion of the first cervical vertebra − increased anterior translation distance in TMD, showing an anteriorization of the cervical spine	“the symptomatic TMD patients presented a flexion of the first cervical vertebra associated with an anteriorization of the cervical spine (hyperlordosis)” “subjects with symptomatic TMD had a tendency to present flexion of the first cervical vertebra and an anteriorization (hyperlordosis) of the cervical spine (C2–C7)”
Di Giacomo P et al., 2018 [17]	CS	59 subjects with skeletal class II 38 females; 21 males study group: 26 patients with TMD control group: 33 patients without TMD	33.65 years average	lateral cephalograms cervical spine analysis clinical diagnosis of TMD (RDC/TMD)	to assess changes in the craniocervical structure and hyoid bone position	− craniocervical angle measurement was out of standard in 40% of subjects with TMD − craniocervical angle in patients with no TMD showed anomalies in 20 of 33 subjects (61%), and in those with TMD it was altered in 13 of 26 subjects (50%) − hyoid bone position was altered in 54% patients with TMD and in 45% of subjects with no TMD	“the significant relationship between skeletal Class II and cervical spine cannot be highlighted” “the alteration of craniocervical angle seems to be mildly present, with backward counterclockwise rotation of the head upon the neck in the sample group” “neck posture could be the result of a compensatory/antalgic mechanism in response to TMD”
Flores HF et al., 2016 [28]	CS	102 patients with TMD (28 men and 74 women) control: 99 subjects without TMD (65 men and 34 women)	study group: mean age 28.93 years (±14.9) control group: mean age 29.32 years (±15.19)	clinical examination RDC/TMD lateral skull and cervical teleradiography biomechanical craniocervical analysis depth of the cervical skull curvature quantitative analysis of the morphometry of the cervical vertebrae	possible relationships between various craniocervical parameters and TMD	in TMD: − altered occipito-atlanto-occipital space − decreased craniovertebral angle − altered depth of the cervical spine − rectified spine − altered hyoid triangle − craniovertebral deformity − altered morphometry of the cervical vertebra	“there is a relationship between the anatomical and functional parameters of the cervical spine in patients with TMD”
John ZAS et al., 2010 [29]	CS	75 cases, 25 cases in each group of class I, II vertical and II horizontal	18–30 years	MRI	− to compare articular disk position, condylar position and joint spaces − to assess the potential for development of TMDs in the 3 groups	alterations in the TMJ morphology in class II vertical and class II horizontal cases, with maximum discrepancy in class II vertical cases	“class II vertical cases are more susceptible to the development of TMDs” “class II vertical cases showed maximum alterations in the disk position, condylar position, and joint spaces.” “there was a tendency for anterior and medial DD with more anteriorly positioned condyles compared with other groups”
Jung WS, et al., 2013 [30]	CS	460 adult patients (117 males and 343 females) skeletal class I, II and III malocclusions	male age range: 18.1–37.8 years (mean 22.7 ± 5.8), female: 17.0–47.3 years (mean 24.1 ± 4.9)	lateral cephalograms MRI 6 groups: N/N; DDR/N; DDR/DDR; DDwR/N; DDR/DDwR; DDwR/DDwR	to analyze the relationships between TMJ DD and skeletal deformities lLinear trends between severity of TMJ DD and sagittal or vertical deformities	the severity of TMJ DD increased as the sagittal skeletal classification changed from skeletal class III to class II and the vertical skeletal classification from hypodivergent to hyperdivergent	“subjects with skeletal class II and/or hyperdivergent deformities have a high possibility of severe TMJ DD” “TMJ DD may be present in patients with various skeletal deformities, regardless of TMJ symptoms”
Kwon HB, et al., 2013 [31]	CS	293 adult patients (80 male and 213 female)	men’s age range: from 18.1 to 37.8 years (mean age 22.7 ± 5.8) women’s age range: from 17.0 to 47.3 years (mean age 24.1 ± 4.9)	lateral cephalogram MRI 3 groups: N/N; DDR/DDR; DDwR/DDwR	to assess gender differences in dentofacial characteristics of adult patients according to TMJ DD	− patients with TMJ DD had short ramus height, short mandibular body length and backward positioning of the ramus and mandible − effective mandibular length even tended to decrease as TMJ DD progressed − male patients showed a larger difference in effective mandibular length between N and DDR − the gonial angle showed no difference between gender or among TMJ DD statuses − overjet was larger in TMD DD	“dentofacial morphology is strongly associated with TMJ DD status” “skeletal Class II hyperdivergent pattern with a short ramus and mandible may be a potential indicator of TMJ DD regardless of gender”
Ma Z et al., 2019 [32]	PS	72 juvenile patients skeletal class II malocclusions	average age: 15.7 years (range: 10–20 years)	MRI DDR	to determine whether ARS can effectively treat TMJ anterior DDR in juvenile class II patients	− functional: wax construction bite − reductions in TMJ pain, TMJ clicking, ROM − improvement of VAS scores for pain and disability in daily life	“ARS is relatively effective in repositioning the DDR, especially for patients in early puberty” “ARS enhances condylar adaptive remodelling and mandibular growth”
Matheus RA, et al., 2018 [33]	CS	60 patients: study group 30 with TMD control group: 30 with no TMD (47 women, 13 men)	mean age 34.2	clinical examination RDC/TMD lateral cephalograms MRI	to evaluate the possibility of any correlation between DD and parameters used for evaluation of skull positioning in relation to the cervical spine: craniocervical angle, suboccipital space between C0-C1, cervical curvature and position of the hyoid bone	− differences were observed between C0–C1 measurement for both symptomatic and asymptomatic − no association between craniocervical angle, C1–C2 and hyoid bone position in relation to DD	“no direct relationship could be determined between the presence of DD and the assessed variables” “there is a close anatomofunctional relationship between the masticatory system and the cervical region and scapular centric” “the postural alteration of the head leads to a disadvantage to muscular biomechanics” “the relationship between craniocervical disorder and TMD may be related to the muscular component rather than the articular one”
Walczynska-Dragon K, et al., 2014 [34]	PS	60 patients with TMD (30 female, 30 male) two groups: with TMD, cervical spine pain and limited cervical spine ROM control group	18–40 years	questionnaire about TMD symptoms and neck pain clinical examination RDC/TMD VAS and the cervical Oswestry scale for the cervical spine pain mandibular motion was recorded by jaw motion analyzer	to evaluate the influence of occlusal splint therapy on cervical spine ROM and spinal pain	occlusal splint therapy showed a significant improvement in TMJ function, cervical spine ROM and a reduction of spinal pain	“there is a significant association between TMD treatment and reduction of cervical spine pain, as far as improvement of cervical spine mobility”

PT-publication type; CS-cross-sectional; PS-prospective study; MRI-magnetic resonance imaging; DDR-disk displacement with reduction; DDwR-disk displacement without reduction; N-normal disk position, TMJ-temporomandibular joint; DD-disk displacement; ID-internal derangement; CVT/EVT-cervical lordosis angle; RDC/TMD-research diagnostic criteria for temporomandibular disorders; ROM-range of movement; VAS-visual analogue scale; ARS-anterior repositioning splint.

**Table 2:** Lateral cephalometric measurements.

Parameter	Value	Mean ± SD	Meaning
SNA	86.5°	82 ± 2°	protruded maxilla
SNB	83°	80 ± 2°	prognathic mandible
*Y* axis to S-N	60°	70 ± 4°	horizontal growth pattern
FMA	11.5°	25 ± 2°	hypodivergent pattern
gonial angle	109°	125 ± 5°	acute gonial angle
occlusal plane to Go-Gn	2.7°	19.09 ± 4.7°	vertical undergrowth of mandible
occlusal plane to S-N	16.3°	14.5 ± 2°	horizontal growth tendency
S-N to Gn	59.5°	67.0 ± 2°	hypodivergent facial pattern
S-N to Go-Me	16.5°	32 ± 2°	horizontal growth tendency
articular angle	141°	145 ± 5°	acute articular angle
facial height ratio	83.48%	65 ± 8%	hypodivergent growth pattern
lower anterior facial height (mm)	98.5 mm	130 ± 3 mm	small anterior facial height
Go-Gn (mandibular plane) to S-N	13.6°	32 ± 4°	hypodivergent facial pattern
Wits appraisal	−0.5 mm	−2.5 ± 0.5 mm	skeletal class II
A-B plane	−7.5 mm	−4.5 ± 2.5 mm	class II malocclusion
overbite	3.5 mm	2 ± 2 mm	normal
overjet	3.5 mm	2 ± 2 mm	normal

S-sella (the center of the sella turcica), N-nasion (the junction of the nasal and frontal bones at the most posterior point on the curvature of the bridge of the nose); A-point A (on the innermost curvature from the maxillary anterior nasal spine to the crest of the maxillary alveolar process); B-point B (the innermost point on the mandible); SNA-angle between Sella-Nasion-A point (sagittal position of the maxilla); SNB-angle between Sella-Nasion-B point (sagittal position of the mandible); FMA-angle between orbitale to porion and point A (Frankfort-mandibular plane angle: facial pattern); Gn-gnathion (the most outward point on the curvature of the symphysis of the mandible); Go-gonion (angles of the mandible); *Y* axis-the line connecting Sella to Gnathion; Me-menton (the lowest point on the symphysis of the mandible); lower anterior facial height: a line between anterior nasal spine and Me; Wits appraisal-difference between perpendiculars from points A and B onto the occlusal plane; SD-standard deviation.
